# Relationship Between SGLT-2i and Ocular Diseases in Patients With Type 2 Diabetes Mellitus: A Meta-Analysis of Randomized Controlled Trials

**DOI:** 10.3389/fendo.2022.907340

**Published:** 2022-05-26

**Authors:** Bin Zhou, Yetan Shi, Rongrong Fu, Haixiang Ni, Lihu Gu, Yuexiu Si, Mengting Zhang, Ke Jiang, Jingyi Shen, Xiangyuan Li, Xing Sun

**Affiliations:** ^1^ Department of General Surgery, HwaMei Hospital, University of Chinese Academy of Sciences, Ningbo, China; ^2^ Ningbo Institute of Life and Health Industry, University of Chinese Academy of Sciences, Ningbo, China; ^3^ The Second Clinical Medical College, Zhejiang Chinese Medical University, Hangzhou, China; ^4^ The First Clinical Medical College, Zhejiang Chinese Medical University, Hangzhou, China; ^5^ The Department of Endocrinology, The First Affiliated Hospital of Zhejiang Chinese Medical University, Hangzhou, China; ^6^ School of Basic Medical Sciences, Zhejiang Chinese Medical University, Hangzhou, China

**Keywords:** SGLT-2i, T2DM, ocular diseases, meta-analysis, RCTs

## Abstract

**Background:**

This meta-analysis was conducted to explore the association between sodium-glucose cotransporter 2 inhibitors (SGLT-2is) and ocular diseases in type 2 diabetes mellitus (T2DM) patients.

**Methods:**

PubMed, Cochrane Central Registry of Controlled Trials, Web of Science and Springer were searched for articles on randomized controlled trials (RCTs) involving T2DM patients treated with SGLT-2i versus placebo or other hypoglycemic agents published prior to August 2021. The primary outcome of this meta-analysis was incidence of ocular diseases, which was assessed using risk ratios (RR) and 95% confidence intervals (CI). We reviewed 47 papers and compared the effect of SGLT-2i with the effect of the control groups (placebo and other hypoglycemic drugs) on the incidence of ocular diseases.

**Results:**

Compared with controls, overall SGLT-2i use in T2DM patients was not associated with incidences of cataract, glaucoma, retinal disease and vitreous disease. Ertugliflozin (RR=0.47, P=0.01) reduced the risk for retinal disease, while empagliflozin (RR=0.44, P=0.05) reduced the risk for diabetic retinopathy (DR) compared with controls. SGLT-2i (RR=0.50, P=0.02), perhaps empagliflozin (RR=0.47, P=0.06), reduced the risk of retinal disease compared with active hypoglycemic agents. Canagliflozin (RR=4.50, P=0.03) increased the risk for vitreous disease compared with placebo.

**Conclusions:**

There was no significant correlation between overall SGLT-2i and ocular diseases (cataract, glaucoma, retinal disease, vitreous disease, corneal disease, conjunctival disease, uveal disease, eye haemorrhage and vision problems) in T2DM patients. Ertugliflozin and empagliflozin may protect against ocular diseases, but canagliflozin may promote ocular diseases.

## Introduction

Currently, diabetes mellitus (DM) and its complications are a global epidemic that pose a serious threat to human health. The prevalence of diabetes for people aged 20-79 years worldwide was 10.5% in 2021 and is expected to reach 12.2% in 2045 (1). Long-term poor control of blood glucose in DM patients affects the large and small arteries and nervous system, leading to serious complications (2), such as cardiovascular disease, diabetic nephropathy, diabetic peripheral neuropathy, diabetic retinopathy (DR) and other diseases (3). DR is the most common microvascular complication of DM involving the eyes, affecting about one-third of adults with DM and is the main cause of blindness (4, 5). DM is also closely related to glaucoma, cataract and other eye diseases, which tend to appear earlier in patients with DM and have a higher probability of impact (6). Studies have shown that intensive glycemic control has an important effect on long-term microvascular complications in patients with type 2 diabetes mellitus (T2DM) and can delay the occurrence and progression of ocular events (7).

A variety of hypoglycemic drugs, including sodium-glucose cotransporter 2 inhibitors (SGLT-2is), are being used either alone or in combination in the clinic to control blood glucose levels. SGLT-2i lower blood glucose levels by inhibiting the reabsorption of glucose by the kidney to produce glycosuria (8). It can also lower body weight and blood pressure (BP), and has significant protective effects on the kidney and cardiovascular system, although it increases the risk of genitourinary infections (8). However, the effect of SGLT-2i on incidences of ocular events compared with other hypoglycemic drugs and/or placebo is unknown. Due to the low incidence of ocular events observed in clinical trials, it is difficult for a single study to have statistical significance. The aim of this study was to carry out a comprehensive and systematic meta-analysis of randomized controlled trials (RCTs) on the effects of SGLT-2i on ocular events in patients with T2DM. We hope that findings from this study can guide future treatment of patients with T2DM.

## Methods

### Literature Search Strategy

This meta-analysis was conducted in accordance with the Preferred Reporting Items for Systematic Reviews and Meta-Analyses guidelines (9). The RCTs included in this study compared the incidences of ocoular diseases between SGLT-2i and placebo or other active hypoglycemic agents in patients with T2DM. Two researchers conducted a systematic and comprehensive search of PubMed, Cochrane Central Registry of Controlled Trials, Web of Science, and Springer databases to seek relevant papers published in English prior to August 2021. The search terms were (Type 2 diabetes OR type 2 diabetes mellitus OR T2DM OR T2D) AND (sodium-glucose cotransporter 2 inhibitor OR SGLT-2i OR sotagliflozin OR janagliflozin OR dapagliflozin OR canagliflozin OR empagliflozin OR ipragliflozin OR tofogliflozin OR ertugliflozin OR luseogliflozin OR sergliflozin OR licogliflozin OR remogliflozin OR bexagliflozin). The two researchers selected the papers to be included in the study by independently searching the articles, scanning the titles and abstracts of the papers, and viewing the full text and supplementary materials. There were no restrictions on the words related to ocular events during retrieval of the articles, to avoid omission of relevant articles.

### Study Selection and Quality Assessment

Studies were included in the meta-analysis based on the following criteria: (1) participants were T2DM patients; (2) RCTs compared the efficacy of SGLT-2i with placebo or other active hypoglycemic drugs; (3) RCTs recorded detailed information about the occurrence of ocular events; (4) follow-up time was ≥12 weeks. Exclusion criteria: (1) duplicate reports; (2) non-English language; (3) data unavailable. Where studies had been updated, the most complete or the most recent paper was included in the analysis. The selected studies were evaluated separately by two researchers, and any differences were discussed and resolved with a third researcher.

The Cochrane Collaboration’s tool was used to evaluate the quality and risk of bias of included RCTs, and to judge from the aspects of sequence generation, allocation concealment, blinding, incomplete outcome data, selective outcome reporting and free of other bias.

### Data Extraction

Two researchers independently extracted the data and a third researcher reviewed it. Data extracted included: (1) basic characteristics of study, for example author, publication year, country, Clinical Trials.gov identifier, inclusion criteria, follow-up time and therapeutic regimen; (2) detailed data of ocular events in the experimental and control groups including the types and number; (3) classification of the lesions according to their occurrence sites, such as retinal diseases (including retinal, macular, optic papillae related diseases), vitreous diseases, corneal diseases, conjunctival diseases, uveal disease (including iris, ciliary body, choroid related diseases), eye haemorrhage and vision problems.

### Statistical Analysis

This meta-analysis was performed using Review Manager 5.3 statistical analysis software. Risk ratios (RR) and 95% confidence intervals (CI) were used to evaluate the count data. I^2^ statistics were used to evaluate the heterogeneity of the included studies. I^2^<30% represented low and I^2^<60% represented moderate heterogeneity. Random effects model was used in this analysis. P ≤ 0.05 was considered to be statistically significant. When more than 10 studies were included, publication bias was evaluated using funnel plots (10) and sensitivity analysis was conducted to evaluate the stability of the results.

## Result

### Study Selection and Study Characteristics

**Figure 1:**
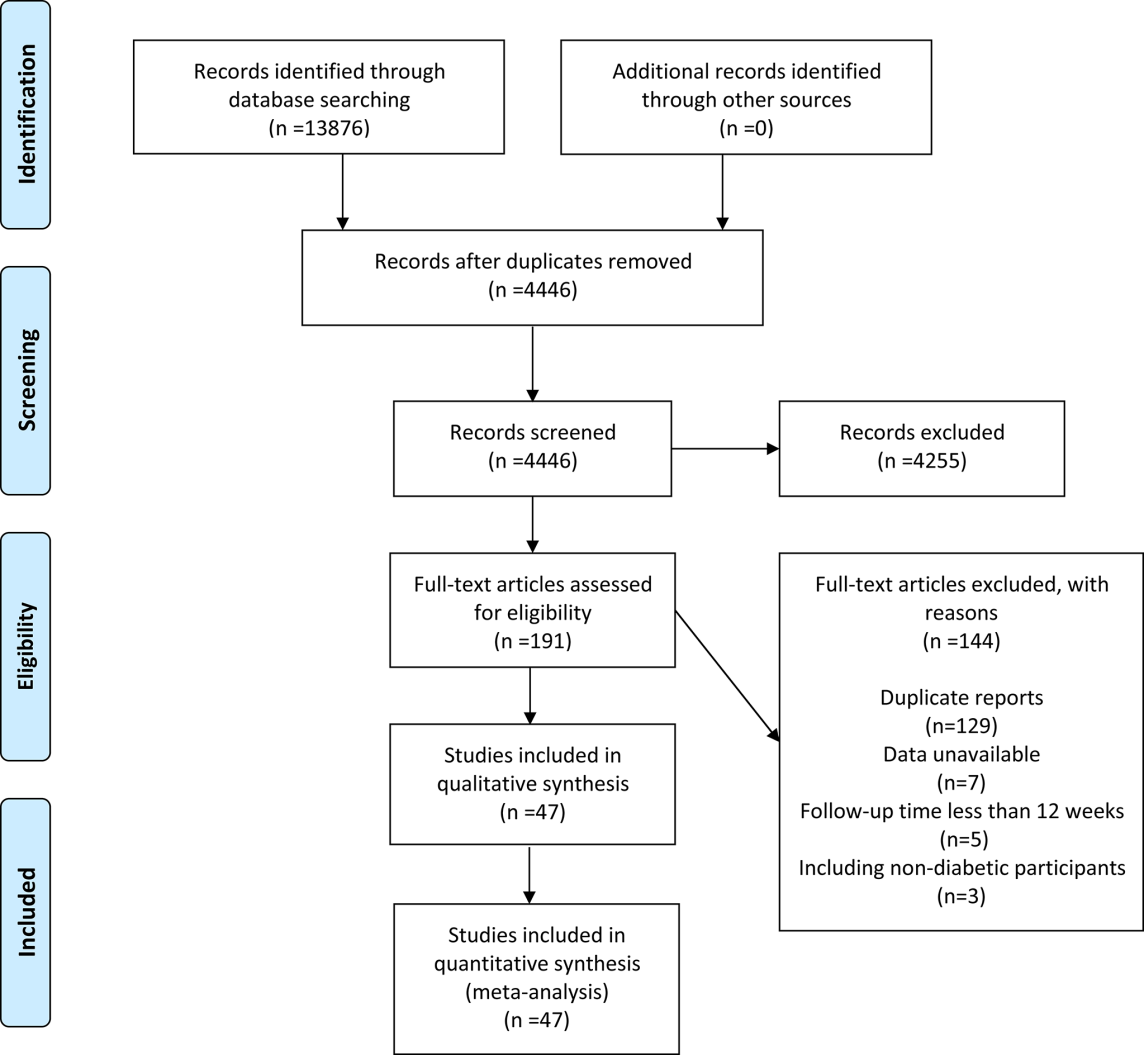
A schematic flow for selecting the articles included in this meta-analysis.

A total of 13876 papers were retrieved, but only 4446 were retained after duplicates were excluded. A total of 4255 articles were removed after reviewing the titles and abstracts. After full-text evaluation of 191 papers, 144 were deleted (129 duplicate reports, 7 data unavailable, 5 follow-up time less than 12 weeks, and 3 included non-diabetic populations). Finally, 47 articles (11–57) (48 RCTs in total) published between 2011 and 2021 were considered (**Figure 1**).

All the studies included in this analysis involved T2DM patients. The experimental group was treated with SGLT-2i intervention (including dapagliflozin, empagliflozin, ertugliflozin, canagliflozin, sotagliflozin, bexagliflozin, tofogliflozin, ipragliflozin), and the control group was treated with placebo or active hypoglycemic drugs (including metformin, sitagliptin, glimepiride, gemigliptin, semaglutide, glipizide, saxagliptin). Follow-up time ranged from 12 to 416 weeks. Most RCTs were multi-country, multi-center studies, with 12 studies conducted in Japan and 1 in Korea (**Table 1**).

**Table 1:** Characteristics of all studies included in the meta-analysis.

Author	Year	Country	Clinical Trials.gov Identifier	Inclusion Criteria	Follow-up time(week)	Therapeutic regimen	
						Experiment	Control
Araki	2016	Japan	NCT02157298	HbA1c 7.2%–11%; INS ≥0.2 IU/kg/day and ≥15 IU/body/day for the past 8 weeks; additional treatment with DPP-4i and eGFR ≥45 ml/min/1.73m^2^	16	Dapagliflozin 5 mg + INS	Placebo + INS
Araki	2015	Japan	NCT01368081	HbA1c 7.0%-10.0%; diet and exercise and monotherapy; no change background antidiabetes therapies for 10 weeks; receive antihypertensive therapy for 4 weeks before randomization	53	Empagliflozin 10 mg/25 mg (SU)	MET (SU)
Aronson	2018	Multicenter	NCT01958671	HbA1c 7.0%-10.5%; diet and exercise; not use OAD (≥8 weeks) or use single OAD	54	Ertugliflozin 5 mg/15 mg	Placebo/MET
Barnett	2014	Multicenter	NCT01164501	HbA1c 7.0%-10.0%; eGFR <90 ml/min/1.73m^2^; diet and exercise; pre-treated with any antidiabetic therapy and no change for 3 months	53	Empagliflozin 10 mg/25 mg	Placebo
Bode	2015	Multicenter	NCT01106651	HbA1c 7.0%-10.0%; FPG <15 mmol/l; patients 55-80 years; no AHA therapy or on stable AHA(s) as monotherapy or combination therapy	104	Canagliflozin 100 mg/300 mg	Placebo
Bolinder	2012	Multicenter	NCT00855166	HbA1c 6.5%-8.5%; women 55-75 years (postmenopausal ≥5 years); men 30-75 years; FPG ≤13.2 mmol/l; body weight ≤120 kg; treatment with MET ≥1500 mg/day (≥3 months)	28	Dapagliflozin 10 mg + MET	Placebo + MET
Cannon	2020	Multicenter	NCT01986881	HbA1c 7.0%-10.5%; patients ≥40 years; evidence or a history of atherosclerosis	312	Ertugliflozin 5 mg/15 mg	Placebo
Cherney	2021	Multicenter	NCT03242018	HbA1c 7%-11%; eGFR 15-30 ml/min/1.73m^2^; drug naive or on AHA(s)	60	Sotagliflozin 200 mg/400 mg	Placebo
Ferrannini	2013	Multicenter	NCT00881530	HbA1c 7.0%-10.0%; drug naive or MET ≥1500 mg/day or maximum tolerated dose ≥10 weeks	79	Empagliflozin 10 mg/25 mg	MET
						Empagliflozin 10 mg/25 mg + MET	Sitagliptin 100 mg + MET
Gallo	2019	Multicenter	NCT02033889	HbA1c 7.0%-10.5%; MET (<8 weeks) or change diabetes regimen	106	Ertugliflozin 5 mg/15 mg	Placebo/Glimepiride
Grunberger	2018	Multicenter	NCT01986855	HbA1c 7.0%–10.5%; diet and exercise with or without AHA monotherapy or combination therapy using other AHAs (INS and SU); eGFR 30-60 ml/min/1.73m^2^	54	Ertugliflozin 5 mg/15 mg	Placebo
Halvorsen	2020	Multicenter	NCT02390050	Treatment-naive: HbA1c 7.0%-8.5%; one OAD: HbA1c 6.5%-8.5%; medications for hypertension or hyperlipidemia must be stable (≥1 month)	12	Bexagliflozin 5 mg/10 mg/20 mg	Placebo
Hollander	2019	Multicenter	NCT01999218	HbA1c 7.0%-9.0%; MET monotherapy ≥1500 mg/day (≥8 weeks)	106	Ertugliflozin 5 mg/15 mg	Glimepiride
Ikeda	2015	Multicenter	NCT00800176	HbA1c 7.0%-10.0%; diet and exercise or with stable MET (≥3 months)	12	Tofogliflozin 2.5 mg/5 mg/10 mg/20 mg/40 mg	Placebo
Inagaki	2016	Japan	NCT02220920	HbA1c 7.5%-10.5%; diet and exercise or with stable INS ≥12 weeks	16	Canagliflozin 100mg + INS	Placebo + INS
Inagaki	2014	Japan	NCT01413204	HbA1c 7.0%-10.0%; diet and exercise ≥55 days or with AHA(s)	26	Canagliflozin 100 mg/200 mg	Placebo
Inagaki	2013	Japan	NCT01022112	HbA1c 6.9%-9.9%; diet and exercise or with AHA(s); no change regimen ≥8 weeks	14	Canagliflozin 50 mg/100 mg/200 mg/300 mg	Placebo
Inzucchi	2021	Multicenter	NCT01289990	HbA1c 7.0%-11%; diet and exercise, drug-naive or pre-treated with pioglitazoneor or with MET or with MET plus SU at 12 weeks	77	Empagliflozin 10 mg (Drug naive)	Placebo (Drug naive)
						Empagliflozin 25 mg (Drug naive)	Sitagliptin 100 mg (Drug naive)
						Empagliflozin 10 mg/25 mg (Pioglitazone)	Placebo (Pioglitazone)
						Empagliflozin 10 mg/25 mg (MET)	Placebo (MET)
						Empagliflozin 10 mg/25 mg (MET + SU)	Placebo (MET + SU)
Ji	2019	Multicenter	NCT02630706	MET (≥1500 mg/day): HbA1c 7.0%-10.5%; MET <1500 mg/day: HbA1c 7.5%-11.0%; dual combination therapy with MET + SU, DDP-4i, meglitinide, or AGI: HbA1c 6.5%-9.5%	28	Ertugliflozin 5 mg/15 mg	Placebo
Kadowaki	2015	Japan	NCT01193218	Diet and exercise; drug naive HbA1c 7.0%-10.0%; one AHA: HbA1c 6.5%-9.0%; Visit 2: HbA1c 7.0%-10%	12	Empagliflozin 5 mg/10 mg/25 mg/50 mg	Placebo
Kashiwagi	2015	Japan	NCT01135433	HbA1c 7.4%-9.9%; MET ≥6 weeks	24	Ipragliflozin 50 mg + MET	Placebo + MET
Kawamori	2018	Japan	NCT02453555	Diet and exercise and with treatment-naive or one OAD ≥12 weeks; treatment-naive: HbA1c 8.0%-10.5%; OAD-pretreated (except linagliptin): HbA1c 7.5%-10.5%; linagliptin-pretreated: HbA1c 7.5%-10.0%	53	Empagliflozin 10 mg/25 mg + Linagliptin 5 mg	Placebo + Linagliptin 5 mg
Kitazawa	2020	Japan	UMIN000026161	HbA1c 7.0%-9.0%; with MET and a DPP-4i	24	Tofogliflozin 20 mg	Glimepiride 0.5 mg
Kitazawa	2021	Japan	jRCTs031180205	HbA1c 6.5%-10.0%; diet and exercise or with OADs; MET ≥500 mg/day with or without an AGI or thiazolidines for ≥8 weeks	52	Ipragliflozin 50 mg + MET	Sitagliptin 50 mg + MET
Kohan	2014	Multicenter	NCT00663260	HbA1c 7.0%-11.0%; eGFR 30-59 ml/min/1.73 m^2^; diet and exercise or with a regimen of any approved AHAs, no change for 6 weeks	28	Dapagliflozin 5 mg/10 mg	Placebo
Kwak	2020	Korea	NCT03202563	HbA1c 7.0%-11.0%; drug naive or with stable MET	12	Dapagliflozin 10 mg	Gemigliptin 50 mg
Lavalle-González	2013	Multicenter	NCT01106677	HbA1c 7%-10.5%; MET therapy ≥2000 mg/day or ≥1500 mg/day for ≥8 weeks; FPG <15 mmol/l at week -2 and fasting fingerstick glucose 6.1-15 mmol/l on day 1	26	Canagliflozin 100 mg	Placebo/Sitagliptin 100 mg
						Canagliflozin 300 mg	Sitagliptin 100 mg
Leiter	2015	Multicenter	NCT00968812	HbA1c 7.0%-9.5%; MET ≥2000 mg/day or ≥1500 mg/day for ≥10 weeks	104	Canagliflozin 100 mg/300 mg	Glimepiride 1-8 mg
Lingvay	2019	Multicenter	NCT03136484	HbA1c 7.0%-10.5%; MET ≥1500 mg/day or maximum tolerated dose for ≥90 days; eGFR ≥60 ml/min/1.73 m²	57	Canagliflozin 300 mg	Semaglutide 1 mg
Müller-Wieland	2018	Multicenter	NCT02471404	HbA1c 7.5%-10.5%; MET ≥1500 mg/day (≥8 weeks); C-peptide ≥1.0 ng/ml; FPG ≤15 mmol/l	52	Dapagliflozin 10 mg + MET	Glimepiride 1-6 mg + MET
Nauck	2011	Multicenter	NCT00660907	HbA1c 6.5%-10%; FPG ≤15 mmol/l; C-peptide ≥1.0 ng/ml; MET or MET plus one other OAD, administer up to half-maximal dose (≥8 weeks)	56	Dapagliflozin 2.5 mg/5 mg/10 mg + MET	Glipizide 5 mg/10 mg/20 mg + MET
Perkovic	2019	Multicenter	NCT02065791	HbA1c 6.5%-12.0%; eGFR 30-90 ml/min/1.73 m^2^; maximum tolerated labeled daily dose of an ACEi or ARB (≥4 weeks); UACR >300 mg/g and ≤5000 mg/g	239	Canagliflozin 100 mg	Placebo
Ridderstråle	2018	Multicenter	NCT01167881	HbA1c 7.0%-10.0%; MET IR ≥1500 mg/day, maximum tolerated dose, or maximum dose according to the local label (≥3 months)	208	Empaglifozin 25 mg	Glimepiride 1-4 mg
Rodbard	2019	Multicenter	NCT02863328	HbA1c 7.0%-10.5%; MET ≥1500 mg/day or maximum tolerated	52	Empagliflozin 25 mg	Semaglutide 14 mg
Rosenstock	2015	Multicenter	NCT01011868	HbA1c 7.0%-10%; INS with or without MET and/or SU (≥3 months)	82	Empagliflozin 10 mg/25 mg	Placebo
Rosenstock	2014	Multicenter	NCT01306214	HbA1c 7.5%-10%; diet and exercise; treatment with INS or with MET	52	Empagliflozin 10 mg/25 mg	Placebo
Rosenstock	2019	Multicenter	NCT02681094	HbA1c 7.5%-10.0%; MET ≥1500 mg/day (≥8 weeks); FPG ≤15 mmol/l	28	Dapagliflozin 5 mg + Saxagliptin 5 mg + MET	Saxagliptin 5 mg + MET
						Dapagliflozin 5 mg + MET	
Ross	2015	Multicenter	NCT01649297	HbA1c 7.0%-10%; diet and exercise; MET ≥1500 mg/day (≥3 months)	17	Empagliflozin 12.5 mg BID/25 mg QD	Placebo
						Empagliflozin 5mg BID/10 mg QD	
Sone	2020	Japan	NCT02589639	INS with or without an OAD (≥3 months); C-peptide >0.5 ng/ml; INS alone: HbA1c 7.5%-10.0%; INS with an OAD: HbA1c 7.0%-9.5%	53	Empagliflozin 10 mg/25 mg	Placebo
Tanaka	2020	Japan	UMIN000024502	HbA1c 6.0%-10.0%; stable glucose-lowering medications ≥1 month; history of established CV disease, including HF	24	Empagliflozin	Placebo
Wilding	2012	Multicenter	NCT00673231	HbA1c 7.5%-10.5%; INS ≥30 U/day (≥8 weeks) or with up to 2 OADs; MET ≥1500 mg/day or maximum tolerated dose and other OADs on at least half the daily maximum dose; diet and exercise	28	Dapagliflozin 2.5 mg/5 mg/10 mg + INS	Placebo + INS
Wiviott	2019	Multicenter	NCT01730534	HbA1c 6.5%-12%; ≥40 years; high risk for CV events; creatinine clearance ≥60 ml/min	270	Dapagliflozin 10 mg	Placebo
Yale	2014	Multicenter	NCT01064414	HbA1c 7.0%-10.5%; eGFR 30-50 ml/min/1.73 m^2^; not on AHA therapy or AHA monotherapy or combination therapy; CKD 3, have generally stable renal function	52	Canagliflozin 100 mg/300 mg	Placebo
Yang	2018	Multicenter	NCT02096705	HbA1c 7.5%-11.0% during screening/enrolment; HbA1c 7.5%-10.5% 14 days prior to randomization; INS ≥20 IU (≥8 weeks)	28	Dapagliflozin 10 mg + INS	Placebo + INS
Yang	2016	Multicenter	NCT01095666	HbA1c 7.5%-10.5%; MET ≥1500 mg/day (≥8 weeks)	24	Dapagliflozin 5 mg/10 mg + MET	Placebo + MET
Zhou	2019	Multicenter	NCT01032629	HbA1c 7.0%-10.5%; ≥30 years with history of CV event, or ≥50 years old with high risk of CV events; not on diabetes drug therapy or on therapy with any approved class of diabetes drugs	416	Canagliflozin 100 mg/300 mg	Placebo
		Multicenter	NCT01989754	HbA1c 7.0%-10.5%; ≥30 years with history of CV event, or ≥50 years old with high risk of CV events; not on AHA therapy, or on AHA monotherapy, or combination AHA therapy	156	Canagliflozin 100-300 mg	Placebo
Zinman	2015	Multicenter	NCT01131676	No glucose-lowering agents: HbA1c 7%-9%; stable glucose-lowering therapy: HbA1c 7%-10%; CV disease; eGFR ≥30 ml/min/1.73m^2^	260	Empagliflozin 10 mg/25 mg	Placebo

AGI, α-glucosidase inhibitor; DPP-4i, dipeptidyl-peptidase-4 inhibitor; SU, sulphonylurea; MET, metformin; INS, insulin; CKD, chronic kidney disease; AHA, anti-hyperglycaemic agent; OAD, oral anti-diabetic drug; eGFR, estimated glomerular filtration rate; FPG, fasting plasma glucose; QD, once daily; BID, twice daily; ACEi, angiotensin-converting enzyme inhibitor; ARB, angiotensin receptor blocker; UACR, urine albumin to creatinine ratio; HF, heart failure; CV, cardiovascular.

### Risk of Bias Assessment

A total of 41 trials had relatively complete records of sequence generation and allocation concealment, so they were considered to have low risk of bias. Only 7 studies had incomplete information, which made it difficult to clarify the risk of bias in sequence generation and allocation concealment. There were 5 studies with high risk in blinding, and the remaining 43 RCTs were all low risk. There was low risk of bias due to incomplete outcome data and selective outcome reporting in all studies. With respect to free of other bias, all studies were difficult to obtain accurate evaluation, so judgment them unclear (**Supplementary Table 1**).

### Effects of SGLT-2i on Incidences of Ocular Events

**Figure 2:**
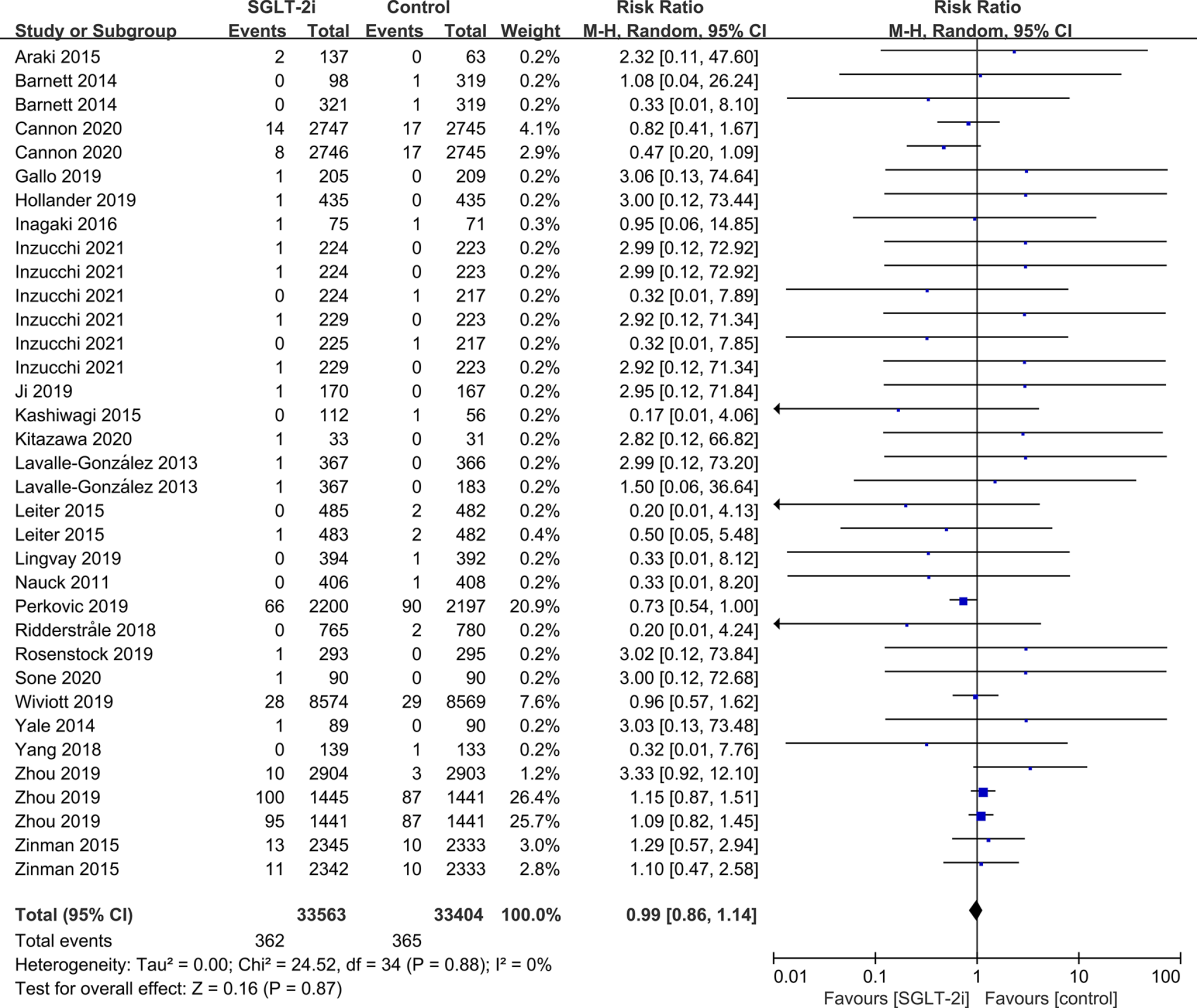
Effect of SGLT-2i on incidences of cataracts compared with control in T2DM patients.

**Figure 3:**
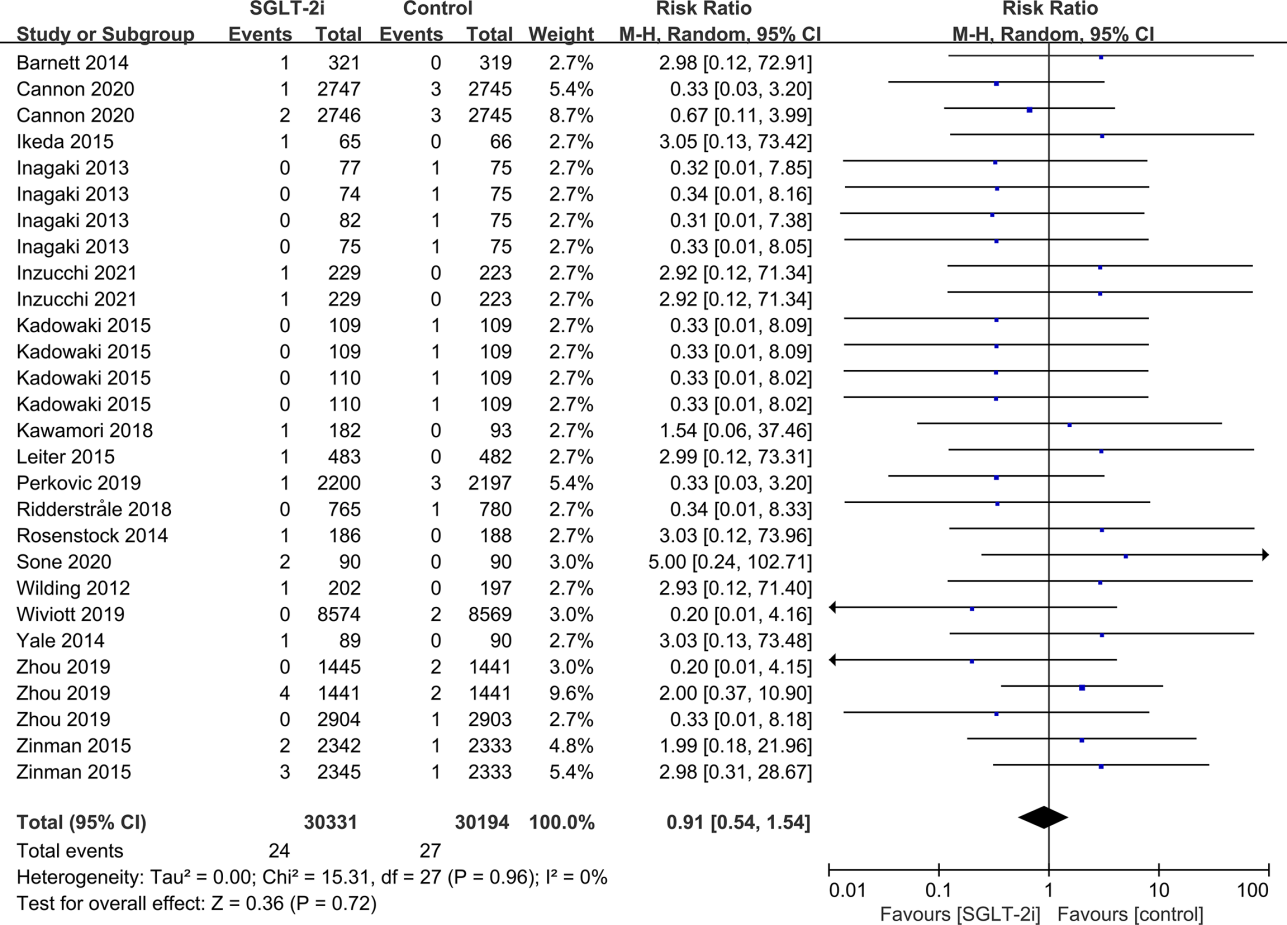
Effect of SGLT-2i on incidences of glaucoma compared with control in T2DM patients.

**Figure 4:**
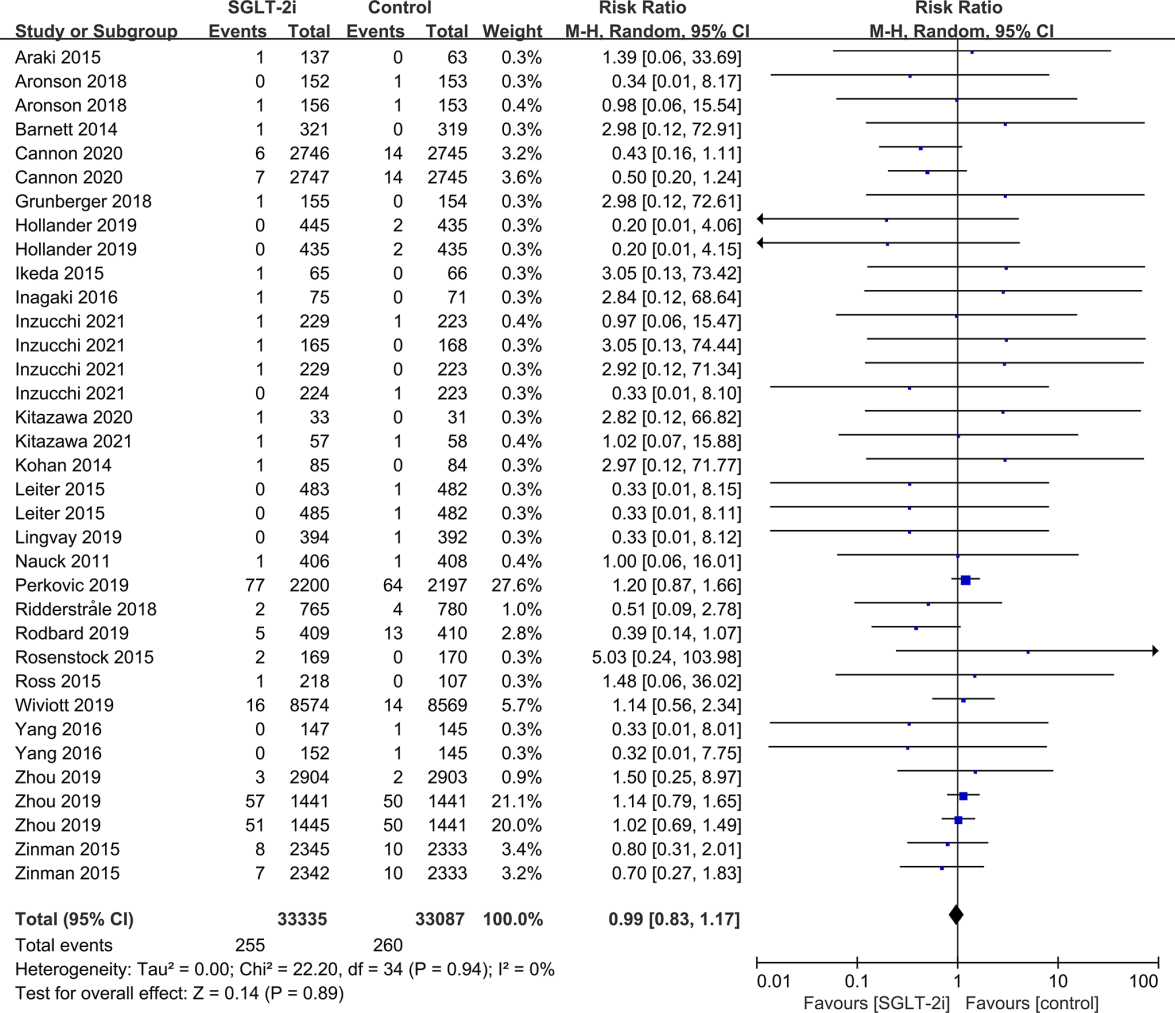
Effect of SGLT-2i on incidences of retinal disease compared with control in T2DM patients.

**Figure 5:**
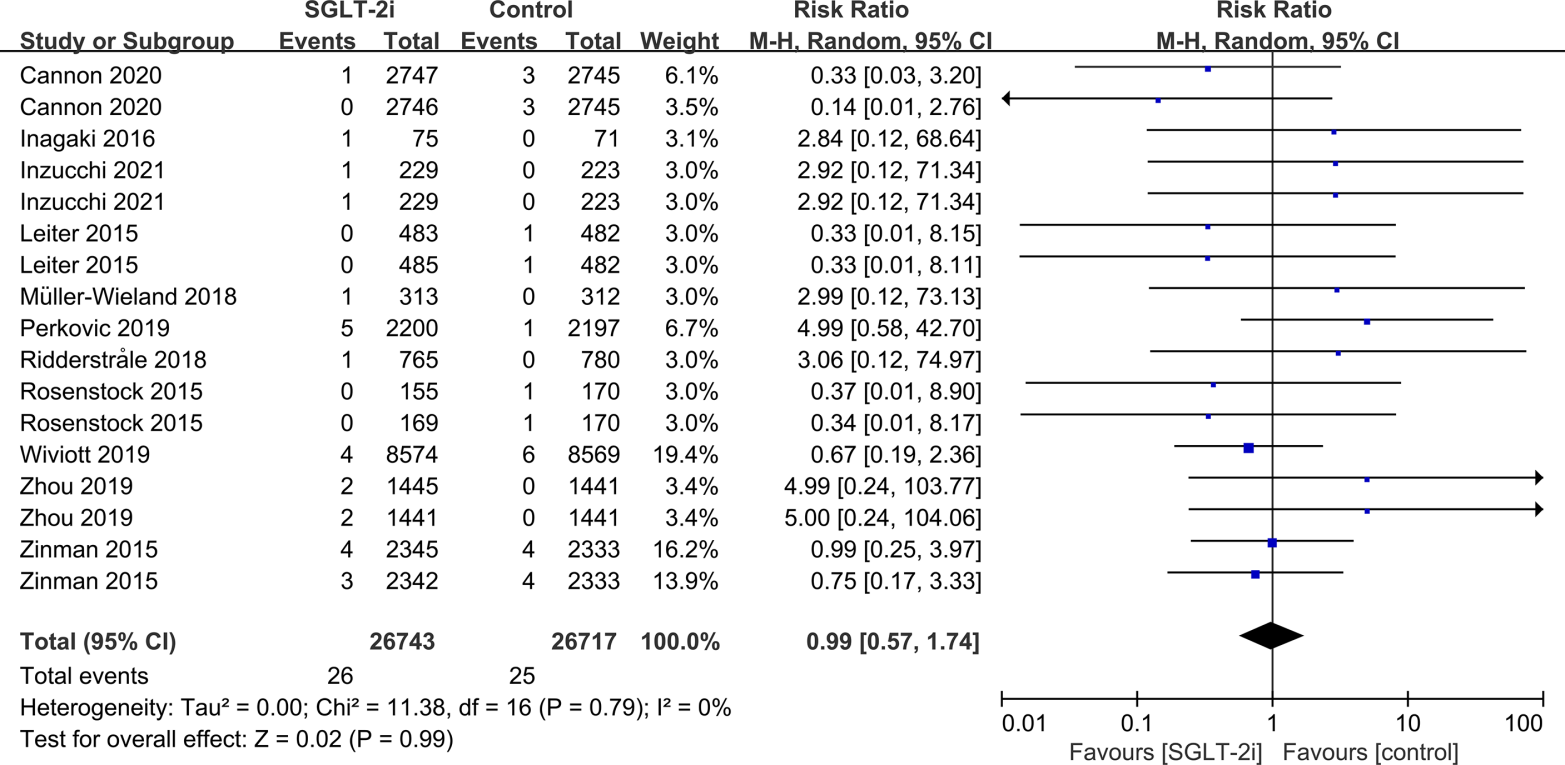
Effect of SGLT-2i on incidences of vitreous disease compared with control in T2DM patients.

SGLT-2i had no effect on cataract risk in T2DM patients compared with the control group (including placebo and/or other active hypoglycemic agents) (RR=0.99, P=0.87) (**Figure 2**). Data were collected from 24 RCTs, with cases of cataracts being reported in 359 cases out of 32743 and in 247 cases out of 25421 patients in the SGLT-2i and control group, respectively. A total of 18 RCTs recorded glaucoma data, including 23 cases out of 30102 patients in the experimental group and 15 cases out of 23123 patients in the control group. The results showed that SGLT-2i had no effect on the risk of glaucoma compared with the control group in T2DM patients (RR=0.91, P=0.72) (**Figure 3**). Analysis of data from 25 RCTs showed that SGLT-2i had no effect on the risk of retinal disease (including retinal, macular, optic papillae related diseases) (RR=0.99, P=0.89), with 254 cases out of 33106 patients and 180 cases out of 25130 patients being reported in the SGLT-2i and control groups, respectively (**Figure 4**). Analysis of data from 11 RCTs showed no difference in incidences of vitreous diseases between the SGLT-2i intervention group and the control group (RR=0.99, P=0.99), with 25 cases out of 26514 patients and 16 cases out of 19546 patients being reported, respectively (**Figure 5**).

Subgroup analysis based on the type of SGLT-2i showed that SGLT-2i had no effect on cataract or glaucoma compared with placebo or active drugs (data not displayed). The risk of cataracts did not change with the type of SGLT-2i (canagliflozin, dapagliflozin, empagliflozin, ertugliflozin) nor the length of follow-up (12-51 weeks, 52-103 weeks, 104-207 weeks, 208 weeks or more) (data not displayed). Similarly, the risk of glaucoma did not change with type of SGLT-2i (empagliflozin, canagliflozin) or length of follow-up (12-51 weeks, 52-207 weeks, 208 weeks or more) (data not shown).

**Figure 6:**
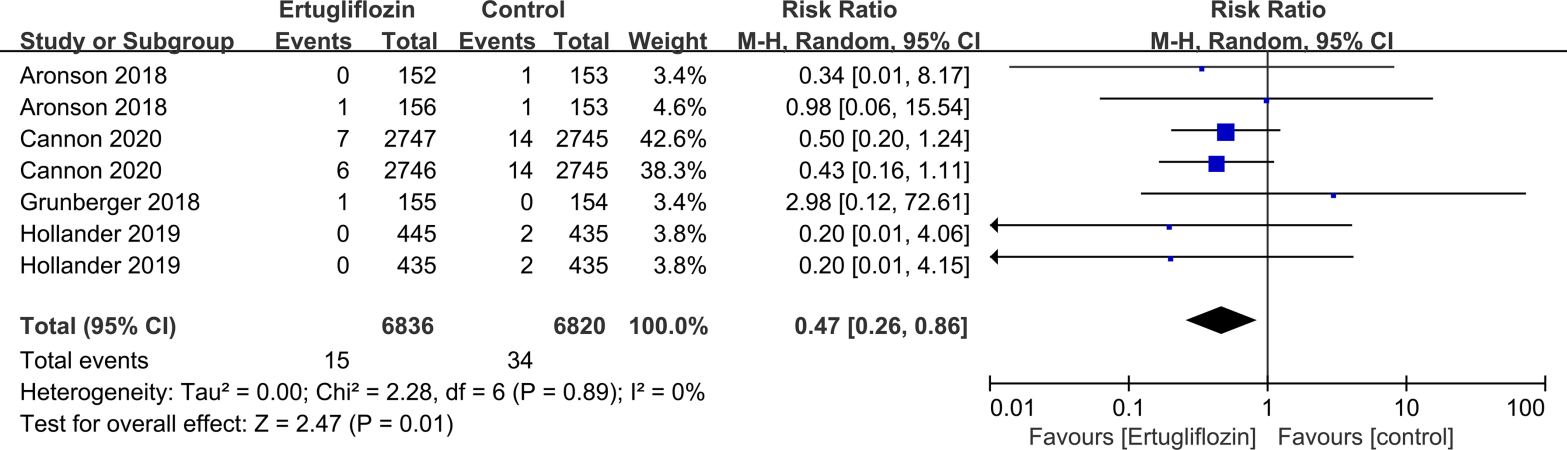
Effect of ertugliflozin on incidences of retinal disease compared with control in T2DM patients.

**Figure 7:**
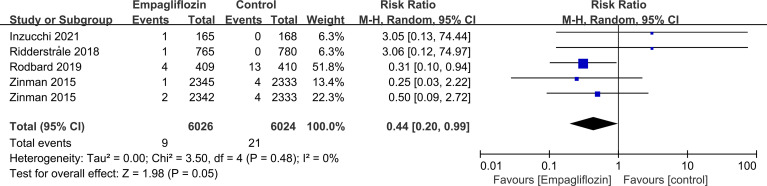
Effect of empagliflozin on incidences of DR compared with control in T2DM patients.

**Figure 8:**
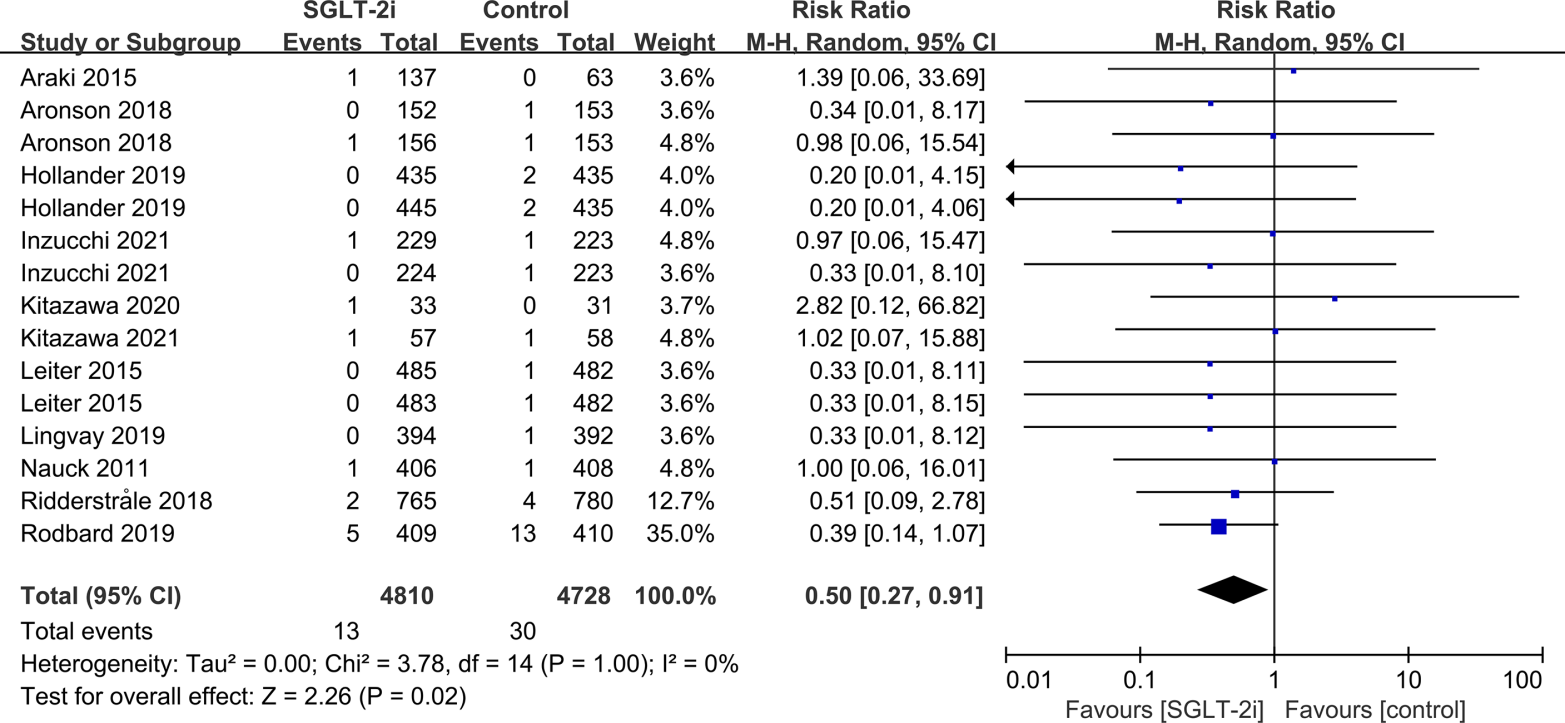
Effect of SGLT-2i on incidences of retinal disease compared with active hypoglycemic agents in T2DM patients.

**Figure 9:**
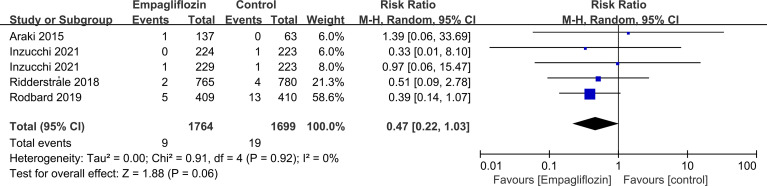
Effect of empagliflozin on incidences of retinal disease compared with active hypoglycemic agents in T2DM patients.

Subgroup analyses of retinal disease based on types of intervention drugs (canagliflozin, dapagliflozin, empagliflozin, ertugliflozin), control drugs (placebo, sulfonylurea) and duration of follow-up (12-51 weeks, 52-103 weeks, 104-207 weeks, 208 weeks or more) showed no effect for the most part (data not shown here), but ertugliflozin was found to significantly reduce the risk of retinal diseases compared with the control group (RR=0.47, 95%CI=0.26 to 0.86, P=0.01) (**Figure 6**). A more targeted analysis of DR found that empagliflozin significantly reduced the risk of DR compared to the control group. This involved 4 studies, with 9 cases out of 6026 people being reported in the empagliflozin group and 17 cases out of 3691 people being reported in the control group (RR=0.44, 95%CI=0.20 to 0.99, P=0.05) (**Figure 7**). SGLT-2i had greater protective effects against retinal diseases compared with active hypoglycemic agents (RR=0.50, 95%CI=0.27 to 0.91, P=0.02) (**Figure 8**), and 4 studies showed that empagliflozin may have a protective effect but not a statistically difference (RR=0.47, 95%CI=0.22 to 1.03, P=0.06) (**Figure 9**).

**Figure 10:**
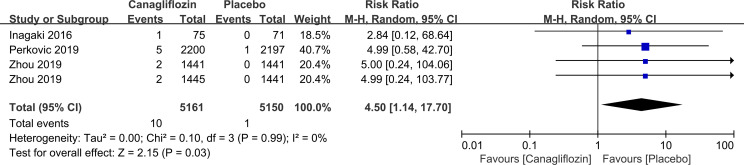
Effect of canagliflozin on incidences of vitreous disease compared with placebo in T2DM patients.

Canagliflozin increased the risk of vitreous disease compared with placebo (RR=4.50, 95%CI=1.14 to 17.70, P=0.03) (**Figure 10**). Another SGLT-2i type (empagliflozin) was not statistically significant compared with placebo (data not displayed).

The use of SGLT-2i in patients with T2DM had no significant effect on corneal disease, conjunctival disease, uveal disease, eye haemorrhage and vision problems compared with the controls (**Figures S1**–**5**). The types of SGLT-2i, control drugs and the length of follow-up time were not associated with the onset (data not shown here).

### Publish Bias and Sensitivity Analyses

Publication bias and sensitivity tests were performed for the effects of SGLT-2i on various ocular events. The funnel plot was roughly symmetrical indicating that there was no significant publication bias. The results were excluded one by one and the sensitivity analysis showed that they were stable.

## Discussion

DM is one of the most serious and common chronic diseases in modern times (1), and is closely related to ocular diseases. Findings from our study showed that SGLT-2i was not associated with incidences of cataracts, glaucoma, retinal disease (including retinal, macular, optic papillae related diseases), vitreous disease, corneal disease, conjunctival disease, uveal disease, eye haemorrhage and vision problems compared with controls in T2DM patients.

Cataracts occur more frequently in DM than in non-diabetics patients, with the prevalence increasing by three- to four-fold in DM patients under 65 years of age and by two-fold in those over 65 years of age (58). Diabetes-induced cataracts develop due to conversion of glucose to polyols by aldose reductase, which leads to an increase in osmotic stress in the lens fibers, causing them to swell and rupture (58). Animal studies have shown that ipragliflozin can delay the progression of cataracts (59). Chen et al. proposed that dapagliflozin might by inhibiting the expression of sodium-glucose cotransporter 2 (SGLT2) and glucose transporters to down-regulate receptor for advanced glycation end products (RAGE) and nicotinamide adenine dinucleotide phosphate (NADPH) oxidases, prevent reactive oxygen species (ROS) accumulation and protect the lens epithelial cells to prevent cataracts in rats with fructose-induced DM (60). In addition, Chin et al. found that T2DM patients treated with metformin had a lower incidence of cataracts than patients not treated with metformin, but it may have something to do with the course of diabetes (61). In our study, we did not observe any correlation between treatment with SGLT-2i and incidences of cataracts, which may be due to the complex mechanisms underlying the development of human cataracts that do not only involve blood glucose levels.

Diabetes may increase the risk of glaucoma in high-risk groups (62), although the underlying mechanism is not completely clear. Some proposed mechanisms include hyperglycemia-induced increase in intraocular pressure by increasing in aqueous humor in anterior chamber and changing trabecular meshwork function, and diabetes-induced microvascular injury and abnormal vascular regulation of optic nerve head and retina which increase the susceptibility to glaucoma injury (63). A retrospective cohort study showed a reduced risk of glaucoma in T2DM patients treated with SGLT-2i compared with patients treated with glucagon-like peptide-1 receptor agonist (GLP-1RA) (64). Due to the limited sample size of this meta-analysis, the data were not enough to compare the incidences of glaucoma between patients treated with SGLT-2i and patients treated with other specific types of hypoglycemic drugs.

In our study, we found that ertugliflozin reduced the risk of retinal disease, and empagliflozin reduced the risk of DR compared to control group. A retrospective cohort study conducted by Su et al. showed that SGLT-2i reduced the risk of diabetic macular edema (DME) in T2DM patients compared with GLP-1RA, through non-hypoglycemic mechanisms (65). Similarly, a study on 3 T2DM patients with chronic DME who were refractory to anti-vascular endothelial growth factor (VEGF) therapy and other treatments found that chronic DME improved after using empagliflozin, dapagliflozin, or luseogliflozin. The exact mechanism of action of SGLT-2i is not clear, but it may be through regulating systemic fluid to improve DME (66). In addition, DME-related symptoms were improved in a T2DM patient with steroid-resistant DME after treatment with ipragliflozin, which is speculated to be related to SGLT-2i’s protection of pericytes from high glucose-induced damage and its direct attenuation of DME by inhibiting VEGF production (67). SGLT-2i is considered to be an alternative for retinal protection in metformin intolerant patients with T2DM (68). A retrospective study showed that treatment with SGLT-2i slowed the progression of DR in patients with T2DM compared with sulfonylurea, independent of its effect on glycemic control (69). However, another study found that SGLT-2i was associated with an increased risk of retinal vein occlusion (RVO), especially in elderly patients and those with chronic kidney disease compared with other hypoglycemic agents (70). Notably, empagliflozin was not linked with retinopathy risk when compared with placebo in the EMPA-REG OUTCOME trial involving T2DM and cardiovascular disease patients (71).

Current studies on the relationship between SGLT-2i and incidences of DR have yielded inconclusive results (72). Long-term hyperglycemia is the basis of the pathogenesis of DR. The initial pathophysiology of DR includes vascular endothelial cell injury and pericytes loss, resulting in hypoxia responses that activate the expression of VEGF and other pro-angiogenic factors, leading to inflammation and tissue dysfunction (73). Hyperglycemia is also associated with defects in red blood cells, which aggravate hypoxia (72). SGLT-2i can reduce glucotoxicity, oxidative stress, inflammation and vascular endothelial dysfunction by reducing glucose in the retinal microcirculation (66). Studies have shown that SGLT2 in retinal pericells may alter cellular tone in response to extracellular glucose concentration. During hyperglycemia, excessive SGLT2 mediates the entry of glucose and sodium in retinal pericytes resulting in change in function and morphology, but this effect can be attenuated by the non-selective sodium-glucose cotransporter (SGLT) inhibitor, phlorizin (74). Herat et al. showed that SGLT-2i can reduce the damage to nerve fibers in the outer layer of the retina by counteracting the overactivation of the sympathetic nervous system (75). A randomized study showed that dapagliflozin has a positive effect on retinal vascular remodeling (76).

Enhanced glycemic control in patients with T2DM has been found to reduce ocular events by 13% (77). In addition, some studies have shown that BP has effect on DR. Analysis of 15 RCTs involving patients with type 1 or 2 diabetes showed that BP control prevented DR for up to 4-5 years (78). Dyslipidemia is also considered a potential risk factor for the progression of DR. Studies have shown that changes in plasma levels of high-density and low-density lipoproteins are closely associated with the severity of DR, and DM patients with dyslipidemia have a higher frequency of retinal abnormalities (73). According to previous studies, SGLT-2i may have a beneficial effect on DR by improving blood glucose, BP and blood lipid in patients with T2DM (79).

In this study, SGLT-2i was found to have a greater protective effect against retinal diseases compared with active hypoglycemic agents. A review by Matuszewski et al. showed that rapid decline in glycaemia would lead to the occurrence and development of DR. The risk of DR was the highest when DM patients were treated with sulfonylurea and insulin, and the lowest when the patients were treated using SGLT-2i, GLP-1RA and dipeptidyl-peptidase-4 inhibitor (DPP-4i) (80). The different mechanisms employed by different drugs to lower blood glucose levels may account for their different effects on retinal diseases. SGLT-2i acts directly on SGLT2 in retinal pericytes, and since SGLT2 is at the beginning of the catastrophic signaling cascade, this pattern may give SGLT-2i unique properties from other antidiabetic agents (79). However, due to the limited sample size, we were unable to conduct a comprehensive statistical analysis of the effects of each class of active hypoglycemic drugs.

Our study found that canagliflozin increased the risk of vitreous disease compared with placebo. Among the studies of T2DM patients using canagliflozin or placebo, only 3 studies recorded the specific data of vitreous diseases (10 cases out of 5161 patients in canagliflozin group and 1 cases out of 3709 patients in placebo group). Vitreous diseases mainly occurred as vitreous haemorrhage. In the canagliflozin group, 8 out of 10 vitreous diseases were vitreous haemorrhage, while in the placebo group, 1 case was vitreous haemorrhage, with no statistical difference between them. And a previous trial showed no statistical difference in vitreous hemorrhage between patients receiving empagliflozin or placebo (81). Retinal ischemia and hypoxia in DM patients have been shown to enhance the expression of angiogenic factors, resulting in the proliferation of retinal neovascular tissue, causing retinal and vitreous adhesion. Hyperplastic traction and constriction of surrounding fibrous components result in vitreous hemorrhage (82). At present, the effect of SGLT-2i on vitreous diseases has not been determined, and the mechanism of action is not entirely clear.

The strength of our study was the inclusion of more trials in the meta-analysis, compared with a similar meta-analysis by Li et al. (6) which included only 9 randomized placebo-controlled trials. In addition, we divided the control group into placebo and other active hypoglycemic agents, which has more diverse directions, and carried out comprehensive subgroup analysis based on the type of hypoglycemic drugs and eye disease. However, there were limitations to our study. Most data on ocular events were derived from reports on adverse event, and there may have been differences in the diagnosis of ocular diseases between studies. Although the subgroup analysis of different types of drugs was comprehensive, the sample size was very limited due to the low incidence of eye diseases. In addition, the use of different medications for varying durations may affect the reliability of statistics. Anyway, this study comprehensively and systematically summarized the relationship between SGLT-2i and eye diseases in RCTs of T2DM patients. More studies on the effect of SGLT-2i on eye prognosis are needed to validate our results.

## Conclusion

SGLT-2i was not associated with incidences of cataracts, glaucoma, retinal disease (including retinal, macular, optic papillae related diseases), vitreous disease, corneal disease, conjunctival disease, uveal disease (including iris, ciliary body, choroid related diseases), eye haemorrhage and vision problems compared with controls in T2DM patients. Ertugliflozin may reduce the risk of retinal disease, while empagliflozin may reduce the risk of DR compared with the control drugs. SGLT-2i has greater protective effects against retinal diseases compared to other hypoglycemic agents, suggesting that empagliflozin may also have protective effects. However, canagliflozin may increase the risk of vitreous disease compared with placebo.

## Data Availability Statement

The original contributions presented in the study are included in the article/**Supplementary Material**. Further inquiries can be directed to the corresponding author.

## Author Contributions

XS and BZ designed the research process. YTS, KJ and LHG searched the database forcorresponding articles. RRF, HXN and JYS extracted useful information from the articles above. YXS, MTZ and XYL used statistical software for analysis. BZ and YTS drafted the meta-analysis. LHG polished this article. All authors had read and approved the manuscript and ensured that this was the case.

## Conflict of Interest

The authors declare that the research was conducted in the absence of any commercial or financial relationships that could be construed as a potential conflict of interest.

## Publisher’s Note

All claims expressed in this article are solely those of the authors and do not necessarily represent those of their affiliated organizations, or those of the publisher, the editors and the reviewers. Any product that may be evaluated in this article, or claim that may be made by its manufacturer, is not guaranteed or endorsed by the publisher.

## Supplementary Material

Supplementary Figure 1 Effect of SGLT-2i on incidences of corneal disease compared with control in T2DM patients.

Supplementary Figure 2 Effect of SGLT-2i on incidences of conjuctival disease compared with control in T2DM patients.

Supplementary Figure 3 Effect of SGLT-2i on incidences of uveal disease compared with control in T2DM patients.

Supplementary Figure 4 Effect of SGLT-2i on incidences of eye haemorrhage compared with control in T2DM patients.

Supplementary Figure 5 Effect of SGLT-2i on incidences of vision problems compared with control in T2DM patients.

## Abbreviations

SGLT-2is, sodium-glucose cotransporter 2 inhibitors; T2DM, type 2 diabetes mellitus; RCTs, randomized controlled trials; RR, risk ratios; CI, confidence intervals; DR, diabetic retinopathy; DM, diabetes mellitus; SGLT2, sodium-glucose cotransporter 2; RAGE, receptor for advanced glycation end products; NADPH, nicotinamide adenine dinucleotide phosphate; ROS, reactive oxygen species; GLP-1RA, glucagon-like peptide-1 receptor agonist; DME, diabetic macular edema; VEGF, vascular endothelial growth factor; RVO, retinal vein occlusion; SGLT, sodium-glucose cotransporter; BP, blood pressure; DPP-4i, dipeptidyl-peptidase-4 inhibitor.
