# Supplementary figures and images for: Relationship Between SGLT-2i and Ocular Diseases in Patients With Type 2 Diabetes Mellitus: A Meta-Analysis of Randomized Controlled Trials
Source: Front Endocrinol (Lausanne). 2022 May 26;13:907340. doi: 10.3389/fendo.2022.907340 (PMC9178099; doi:10.3389/fendo.2022.907340)

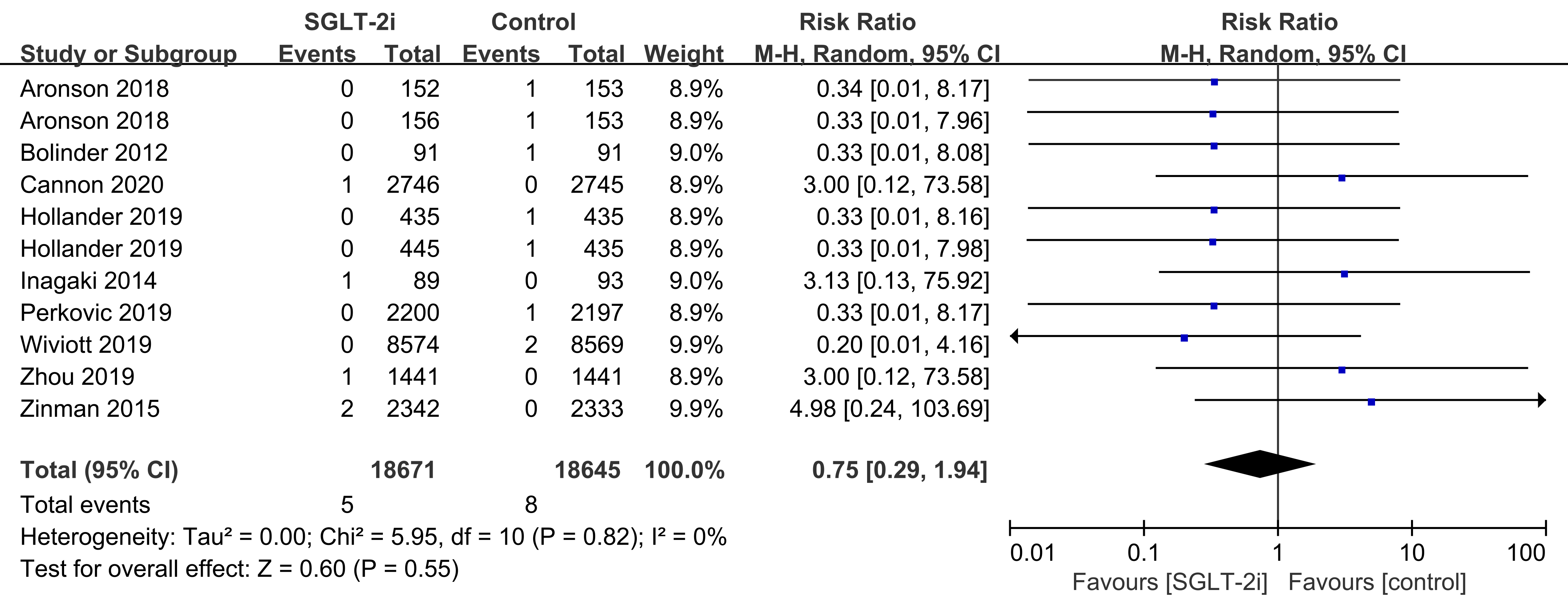

Supplement: Supplementary Figure 1 — Effect of SGLT-2i on incidences of corneal disease compared with control in T2DM patients. [file Image_1.tif]

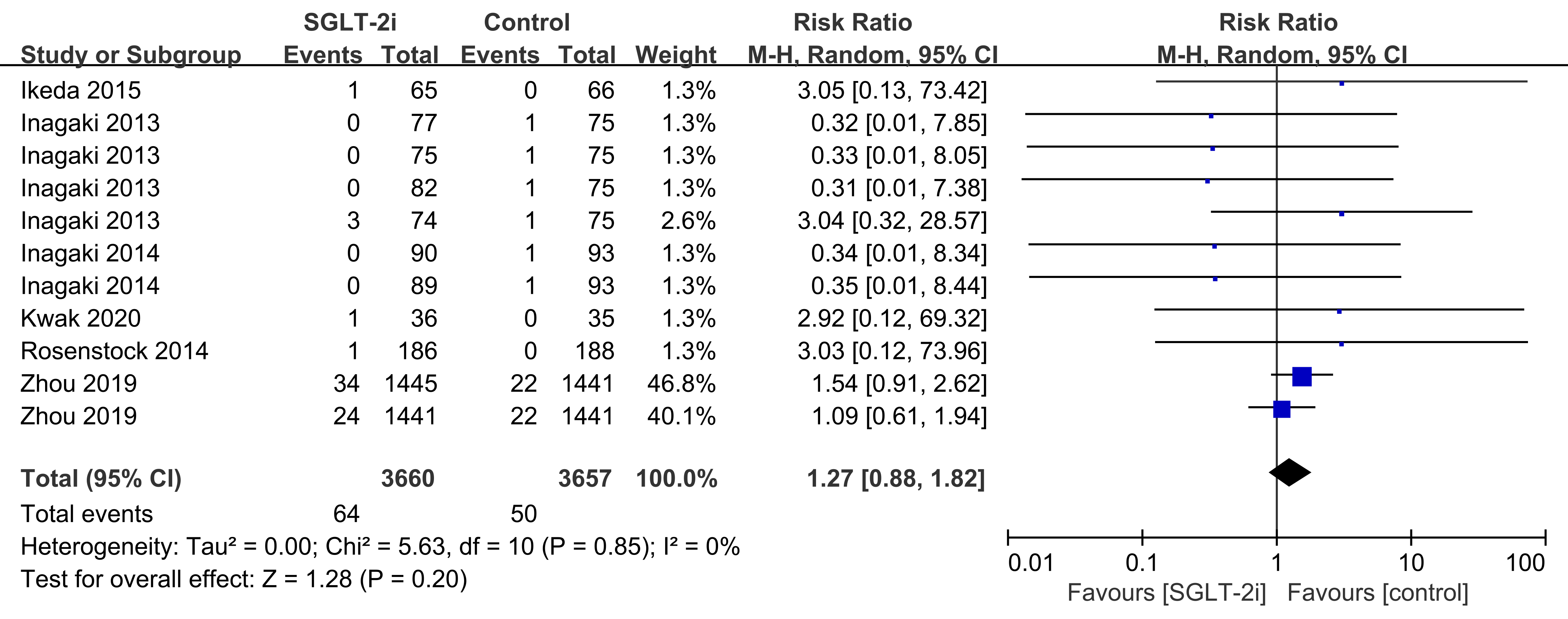

Supplement: Supplementary Figure 2 — Effect of SGLT-2i on incidences of conjuctival disease compared with control in T2DM patients. [file Image_2.tif]

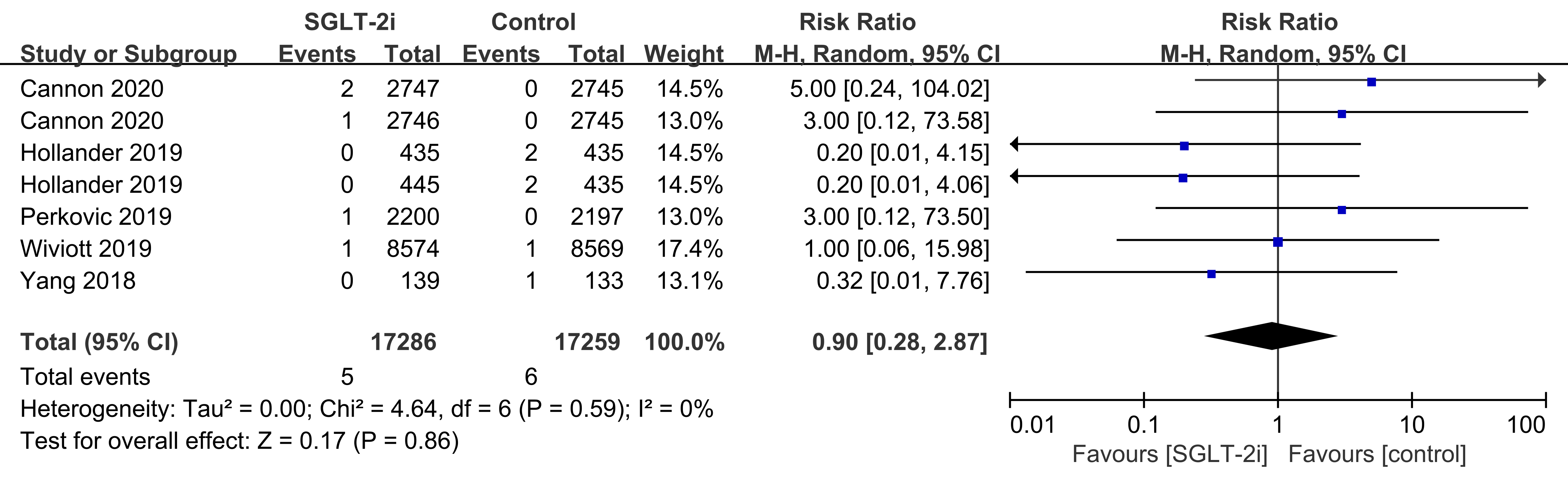

Supplement: Supplementary Figure 3 — Effect of SGLT-2i on incidences of uveal disease compared with control in T2DM patients. [file Image_3.tif]

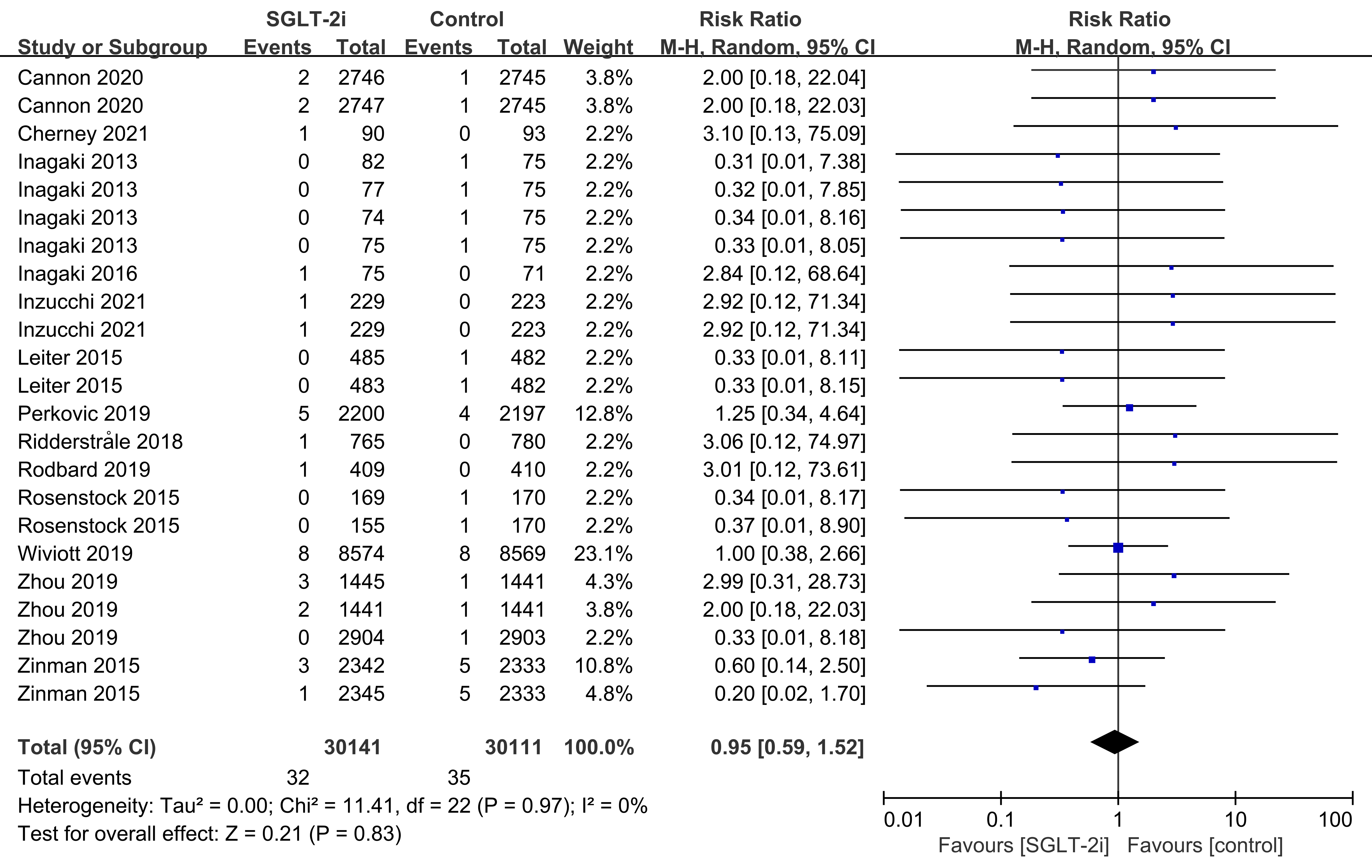

Supplement: Supplementary Figure 4 — Effect of SGLT-2i on incidences of eye haemorrhage compared with control in T2DM patients. [file Image_4.tif]

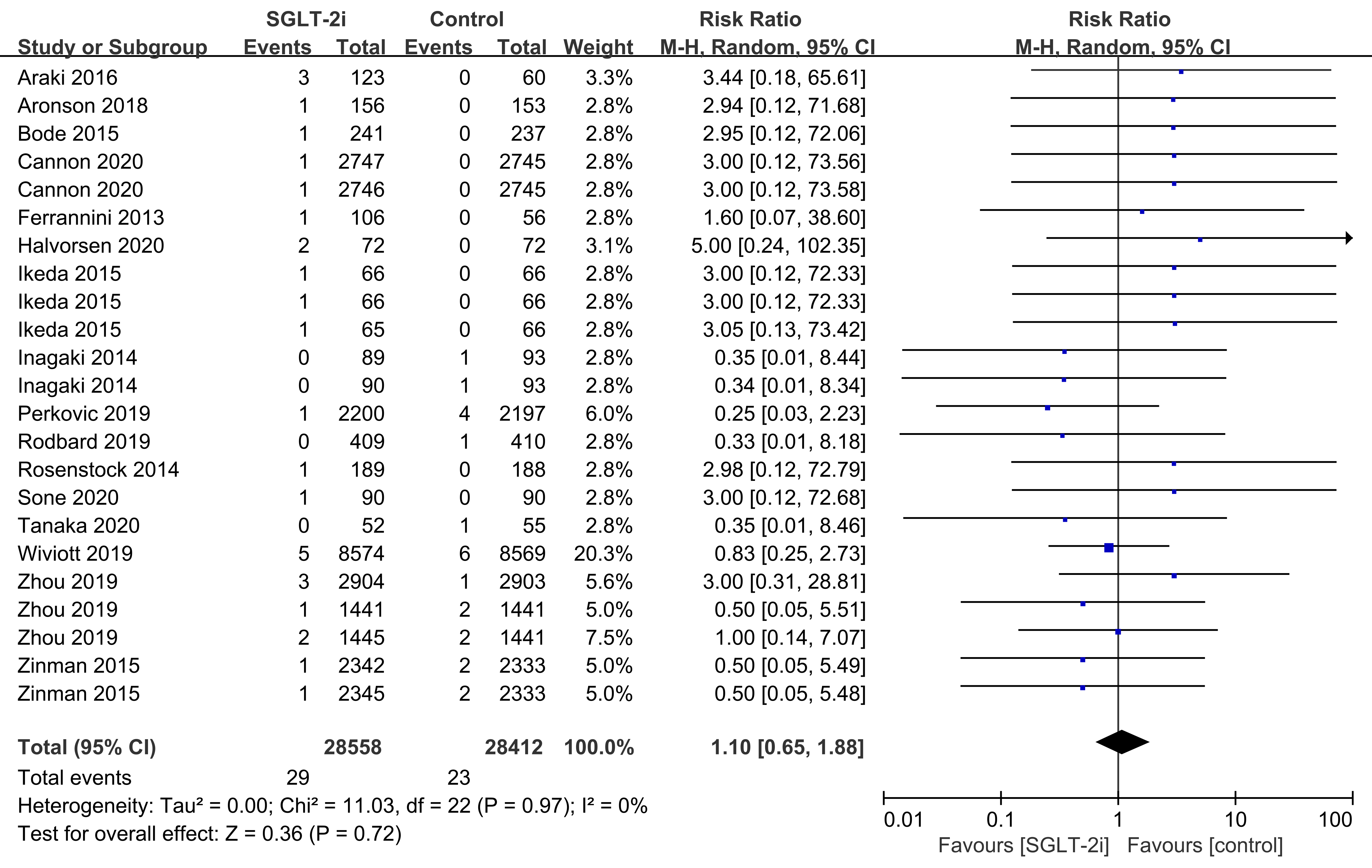

Supplement: Supplementary Figure 5 — Effect of SGLT-2i on incidences of vision problems compared with control in T2DM patients. [file Image_5.tif]
