# Supplementary material for: Relationship Between SGLT-2i and Ocular Diseases in Patients With Type 2 Diabetes Mellitus: A Meta-Analysis of Randomized Controlled Trials
Source: Front Endocrinol (Lausanne). 2022 May 26;13:907340. doi: 10.3389/fendo.2022.907340 (PMC9178099; doi:10.3389/fendo.2022.907340)
Supplement: Supplementary file 6 [file Table_1.docx]

**Supplementary Table 1.** Quality assessment of studies included.

| Author, year | Sequence generation | Allocation concealment | Blinding | Incomplete outcome data | Selective outcome reporting | Free of other bias |
| --- | --- | --- | --- | --- | --- | --- |
| Araki, 2016 | unclear risk | unclear risk | low risk | low risk | low risk | unclear risk |
| Araki, 2015 | low risk | low risk | low risk | low risk | low risk | unclear risk |
| Aronson, 2018 | low risk | low risk | low risk | low risk | low risk | unclear risk |
| Barnett, 2014 | low risk | low risk | low risk | low risk | low risk | unclear risk |
| Bode, 2015 | low risk | low risk | low risk | low risk | low risk | unclear risk |
| Bolinder, 2012 | low risk | low risk | low risk | low risk | low risk | unclear risk |
| Cannon, 2020 | low risk | low risk | low risk | low risk | low risk | unclear risk |
| Cherney, 2021 | unclear risk | unclear risk | low risk | low risk | low risk | unclear risk |
| Ferrannini, 2013 | low risk | low risk | high risk | low risk | low risk | unclear risk |
| Gallo, 2019 | low risk | low risk | low risk | low risk | low risk | unclear risk |
| Grunberger, 2018 | low risk | low risk | low risk | low risk | low risk | unclear risk |
| Halvorsen, 2020 | low risk | low risk | low risk | low risk | low risk | unclear risk |
| Hollander, 2019 | low risk | low risk | low risk | low risk | low risk | unclear risk |
| Ikeda, 2015 | unclear risk | unclear risk | low risk | low risk | low risk | unclear risk |
| Inagaki, 2016 | low risk | low risk | low risk | low risk | low risk | unclear risk |
| Inagaki, 2014 | low risk | low risk | low risk | low risk | low risk | unclear risk |
| Inagaki, 2013 | low risk | low risk | low risk | low risk | low risk | unclear risk |
| Inzucchi, 2021 | low risk | low risk | low risk | low risk | low risk | unclear risk |
| Ji, 2019 | low risk | low risk | low risk | low risk | low risk | unclear risk |
| Kadowaki, 2015 | low risk | low risk | low risk | low risk | low risk | unclear risk |
| Kashiwagi, 2015 | unclear risk | unclear risk | low risk | low risk | low risk | unclear risk |
| Kawamori, 2018 | low risk | low risk | low risk | low risk | low risk | unclear risk |
| Kitazawa, 2020 | low risk | low risk | high risk | low risk | low risk | unclear risk |
| Kitazawa, 2021 | low risk | low risk | high risk | low risk | low risk | unclear risk |
| [Kohan, 2014](https://pubmed.ncbi.nlm.nih.gov/?sort=date&term=Kohan+DE&cauthor_id=24067431) | unclear risk | unclear risk | low risk | low risk | low risk | unclear risk |
| Kwak, 2020 | low risk | low risk | high risk | low risk | low risk | unclear risk |
| Lavalle-González, 2013 | low risk | low risk | low risk | low risk | low risk | unclear risk |
| Leiter, 2015 | low risk | low risk | low risk | low risk | low risk | unclear risk |
| Lingvay, 2019 | low risk | low risk | low risk | low risk | low risk | unclear risk |
| Müller-Wieland, 2018 | low risk | low risk | low risk | low risk | low risk | unclear risk |
| Nauck, 2011 | low risk | low risk | low risk | low risk | low risk | unclear risk |
| Perkovic, 2019 | low risk | low risk | low risk | low risk | low risk | unclear risk |
| Ridderstråle, 2018 | low risk | low risk | low risk | low risk | low risk | unclear risk |
| Rodbard, 2019 | low risk | low risk | high risk | low risk | low risk | unclear risk |
| Rosenstock, 2015 | low risk | low risk | low risk | low risk | low risk | unclear risk |
| Rosenstock, 2014 | low risk | low risk | low risk | low risk | low risk | unclear risk |
| Rosenstock, 2019 | low risk | low risk | low risk | low risk | low risk | unclear risk |
| Ross, 2015 | unclear risk | unclear risk | low risk | low risk | low risk | unclear risk |
| Sone, 2020 | low risk | low risk | low risk | low risk | low risk | unclear risk |
| Tanaka, 2020 | low risk | low risk | low risk | low risk | low risk | unclear risk |
| Wilding, 2012 | low risk | low risk | low risk | low risk | low risk | unclear risk |
| Wiviott, 2019 | unclear risk | unclear risk | low risk | low risk | low risk | unclear risk |
| Yale, 2014 | low risk | low risk | low risk | low risk | low risk | unclear risk |
| Yang, 2018 | low risk | low risk | low risk | low risk | low risk | unclear risk |
| Yang, 2016 | low risk | low risk | low risk | low risk | low risk | unclear risk |
| Zhou, 2019 | low risk | low risk | low risk | low risk | low risk | unclear risk |
|  | low risk | low risk | low risk | low risk | low risk | unclear risk |
| Zinman, 2015 | low risk | low risk | low risk | low risk | low risk | unclear risk |

The RCTs were assessed by the Cochrane Collaboration’s tool. Risk of bias was assessed as “low risk”, “high risk” or “unclear risk”.
